# A clinical course of a patient with anorexia nervosa receiving surgery for superior mesenteric artery syndrome

**DOI:** 10.1186/s40337-021-00436-2

**Published:** 2021-06-30

**Authors:** Ken Kurisu, Yukari Yamanaka, Tadahiro Yamazaki, Ryo Yoneda, Makoto Otani, Yoshiyuki Takimoto, Kazuhiro Yoshiuchi

**Affiliations:** Department of Stress Sciences and Psychosomatic Medicine, Graduate School of Medicine, The University of Tokyo, 7-3-1, Hongo, Bunkyo-ku, Tokyo, 113-8655 Japan

**Keywords:** Anorexia nervosa, Eating disorder, Superior mesenteric artery syndrome, Surgery, Weight recovery

## Abstract

**Background:**

Superior mesenteric artery (SMA) syndrome is a well-known but relatively rare complication of anorexia nervosa. Although several reports have proposed surgery for SMA syndrome associated with anorexia nervosa, these have shown poor outcomes or did not reveal the long-term weight course. Thus, the long-term effectiveness of surgery for SMA syndrome in such cases remains unclear. This case report describes a patient with anorexia nervosa who underwent surgery for SMA syndrome.

**Case presentation:**

An 18-year-old woman presented with anorexia nervosa when she was 16 years old. She also presented with SMA syndrome, which seemed to be caused by weight loss due to the eating disorder. Nutrition therapy initially improved her body weight, but she ceased treatment. She reported that symptoms related to SMA syndrome had led to her weight loss and desired to undergo surgery. Laparoscopic duodenojejunostomy was performed, but her body weight did not improve after the surgery. The patient eventually received conservative nutritional treatment along with psychological approaches, which led to an improvement in her body weight.

**Conclusions:**

The case implies that surgery for SMA syndrome in patients with anorexia nervosa is ineffective for long-term weight recovery and that conservative treatment can sufficiently improve body weight; this is consistent with the lack of evidence on the topic and reports on potential complications of surgery. Due to difficulties in assessing psychological status, consultation with specialists on eating disorders is necessary for treating patients with severely low body weight.

## Background

Superior mesenteric artery (SMA) syndrome is a disease resulting from the compression of the third part of the duodenum between the aorta and the SMA. A narrowing of the aortomesenteric angle, which is caused by a decrease in fatty tissue around the SMA due to weight loss, results in this compression and causes various abdominal symptoms such as nausea, vomiting, and abdominal pain. Conservative treatment generally improves SMA syndrome, but surgical treatment has also been proposed for severe cases [1, 2].

Weight loss due to anorexia nervosa is a well-known but relatively rare cause of SMA syndrome [3]. Conservative treatment for SMA syndrome associated with anorexia nervosa, including a liquid diet, nasojejunal tube placement, and total parenteral nutrition, is reportedly effective [3–5]. Several reports proposed surgery for SMA syndrome associated with anorexia nervosa, but revealed no weight gain for four months [6] or only reported weight gain for two months [7]. Thus, the long-term effectiveness of surgery for weight gain in such cases remains unclear, requiring investigations of the longer-term weight course after surgery. This case report presents a patient with anorexia nervosa who consequently presented with SMA syndrome and underwent surgery.

## Case presentation

**Fig. 1:**
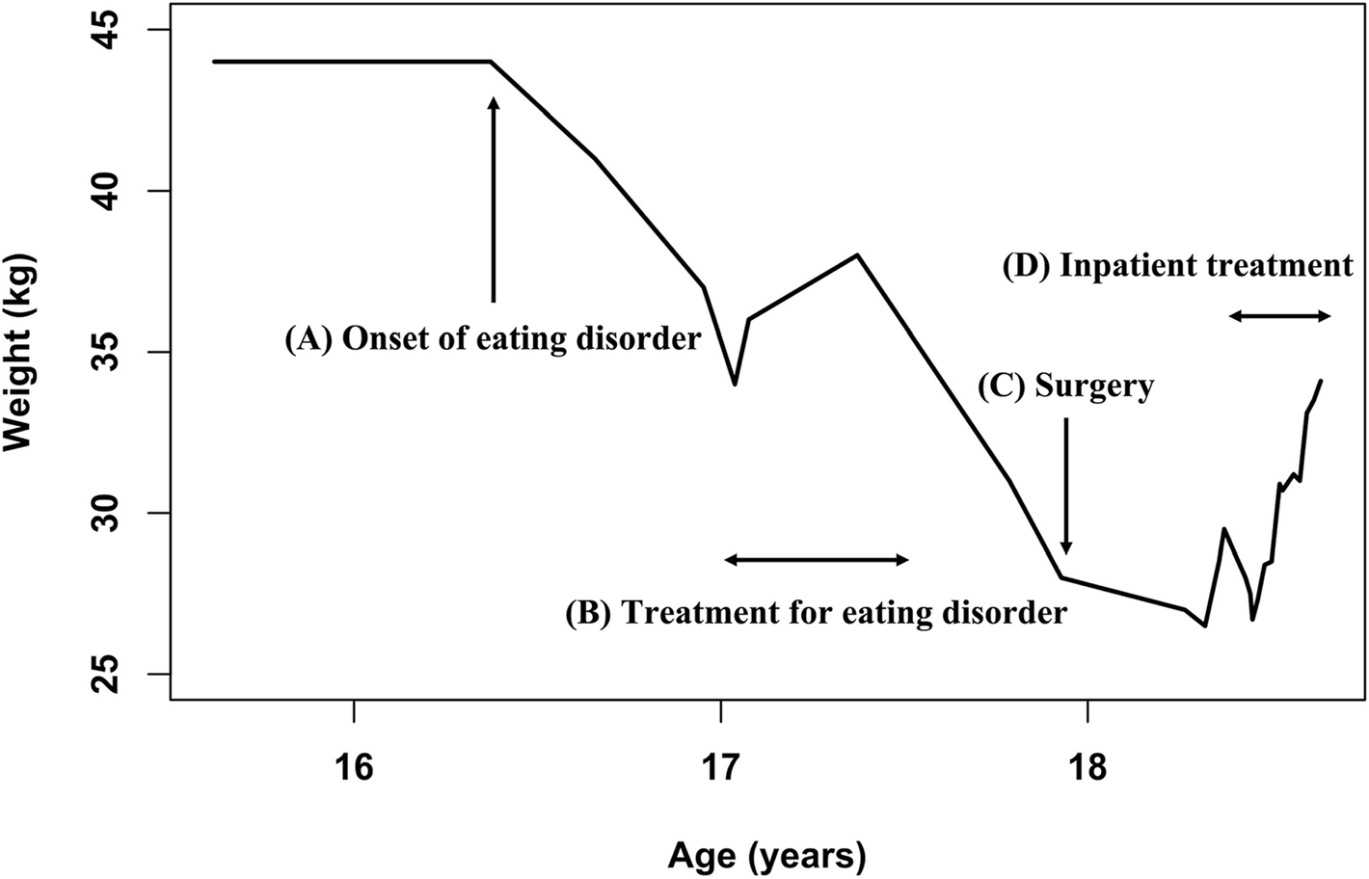
Course of the patient’s body weight. (**A**) The patient’s weight loss started when she was 16 years old. (**B**) Her weight improved during the treatment, which she stopped engaging in later. (**C**) After the surgery for SMA syndrome, her weight did not improve. (**D**) She came to our hospital and received inpatient treatment, which led to an improvement in her body weight

The patient was an 18-year-old Japanese woman with no relevant medical history. Figure 1 shows the course of her body weight, which had been around 44 kg until disease onset. Her weight loss started when she was 16 years old. She was diagnosed with an eating disorder and also presented with SMA syndrome, which seemed to be caused by weight loss due to the eating disorder because no other organic abnormality was present. The patient received conservative nutrition therapy at a hospital located in her residential area when she was 17 years old. The treatment initially improved her weight, but she ceased treatment due to dissatisfaction with the patient–doctor relationship, and her weight loss resumed.

The patient reported that abdominal symptoms due to SMA syndrome, such as abdominal distension and vomiting, resulted in weight loss. She desired to undergo surgery for SMA syndrome, and found on the internet a distant hospital that performed surgery for SMA syndrome. However, after undergoing laparoscopic duodenojejunostomy, her body weight did not improve during the six months after the surgery. When she was 18 years old, she arrived at our center, the University of Tokyo Hospital, with a chief complaint of weight loss. She was immediately hospitalized due to severe malnutrition.

At hospitalization, her height and weight were 159.1 cm and 27.5 kg, respectively (i.e., body mass index [BMI] of 10.9). She was diagnosed with anorexia nervosa binge-eating/purging type (ANBP) according to the Diagnostic Statistical Manual of Mental Disorders, Fifth Edition [8]. Notably, her vomiting, which had been considered a symptom of SMA syndrome before surgery, did not improve after surgery. Therefore, her vomiting could have been a symptom of ANBP, while she initially concealed her self-induced vomiting from us and only revealed it six months after the initial visit to our hospital.

After hospitalization, conservative treatment, including dietary nutrition and central venous nutrition, was performed while she was also monitored for refeeding syndrome. We further performed psychological work with the patient and her family, including psychoeducation, nutritional counseling with registered dietitians, and behavioral management based on operant conditioning [9, 10]. Her body weight gradually improved from 27.5 kg (BMI of 10.9) on day 0 to 34.1 kg (BMI of 13.5) on day 70.

## Discussion and conclusions

We report on a patient with ANBP who consequently presented with SMA syndrome. Nutrition therapy initially improved her body weight, but after ceasing therapy, she underwent laparoscopic duodenojejunostomy, which did not improve her body weight. She subsequently received conservative treatment in our hospital, and her body weight improved. There are several issues regarding this case.

First, the absence of weight gain during the six months after the surgery implies that duodenojejunostomy for SMA syndrome associated with anorexia nervosa may not improve body weight. This implication does not conflict with previous reports that revealed a poor outcome [6] or showed only a short-term outcome for surgery in such cases [7]. This case also implies a poor long-term outcome of this surgery, which would be a new finding.

Second, clinicians should interpret with caution abdominal symptoms and request for surgery by patients with anorexia nervosa. These patients often present with gastrointestinal complications besides SMA syndrome [3]. They also tend to attribute anorexia not with fat phobia but with abdominal symptoms [8, 11], and deny or conceal their symptoms [12, 13]. Notably, this case presented with vomiting, probably as a symptom of ANBP, but the patient reported that SMA syndrome induced the symptom, which might reflect such an attribution [8, 11] or denial [12, 13]. This psychological state, possibly including a desire for people to believe she did not have an eating disorder, might have predisposed her to persuade her parents or doctors to proceed with surgery.

Third, there are several potential complications associated with surgery on patients with anorexia nervosa. Laparoscopic duodenojejunostomy for SMA syndrome can reportedly induce paralytic ileus and leakage [14, 15]. Severe malnutrition is detrimental to the outcome of surgery [16, 17]. Postoperative vomiting induces severe comorbidities such as wound dehiscence [18], and thus, the purging symptom may also induce such clinical problems.

Fourth, the patient’s weight gain in the previous hospital and our center suggests that conservative nutrition therapy with psychological approaches can sufficiently improve body weight. Because SMA syndrome occurs due to low weight [1, 2], it is biologically plausible to cure the syndrome through weight recovery. Conservative treatment could reportedly treat patients with SMA syndrome due to anorexia nervosa [3–5]. Clinicians should sufficiently perform such conservative treatments before proceeding to surgery.

Finally, early consultation with specialists on eating disorders could avoid unnecessary surgery. The assessment of psychological status, including denial or concealment, may require sufficient clinical experience. Establishing a multidisciplinary team is essential for treating anorexia nervosa [9, 10].

In summary, the case implies that surgery for SMA syndrome in patients with anorexia nervosa is ineffective for weight recovery, and conservative treatment along with psychological approaches can sufficiently improve weight. Diagnosis and treatment for young patients with severely low weight and abdominal symptoms should be performed carefully, and consultation with specialists on eating disorders is necessary, especially before proceeding to surgery.

## Data Availability

The datasets are available from the corresponding author on reasonable request.

## Abbreviations

ANBP: Anorexia nervosa binge-eating/purging type
BMI: Body mass index
SMA: Superior mesenteric artery
